# Traditional medicines and their common uses in central region of Syria: Hama and Homs – an ethnomedicinal survey

**DOI:** 10.1080/13880209.2021.1936078

**Published:** 2021-06-24

**Authors:** Chadi Khatib, Abdulhakim Nattouf, Mohamad Isam Hasan Agha

**Affiliations:** a Department of Pharmacognosy, Faculty of Pharmacy, Damascus University, Damascus, Syria; b Department of Pharmaceutics and Pharmaceutical Technology, Faculty of Pharmacy, Damascus University, Damascus, Syria

**Keywords:** Traditional Arabic medicine (TAM), herbal medicine, Mediterranean, phytotherapy, medicinal plants, folk uses, ethnobotanical, ethnopharmacology

## Abstract

**Context:**

Since ancient times, traditional Arabic medicine (TAM) has been used to treat various diseases in Syria. They are cost-effective with fewer side effects and are more suitable for long-term use compared with chemically synthesized medicines. In addition, the scientific importance is manifested, as this survey proceeds, for the purposes of verifying and documenting these traditional medicines and their common uses.

**Objective:**

We conducted ethnobotanical and ethnomedicine research on plants traditionally used to treat various diseases in central region of Syria.

**Methods:**

Information was collected from 2019 to 2021 from the cities of Homs and Hama and their villages, which are two governorates located in central Syria, after interviews with traditional practitioners called *Attarin*, and many other people. Plant specimens were collected according to different references concerning medicinal plants of Syria, to document the traditional uses of plants at least two of the traditional healers and three other people were asked.

**Results:**

In this survey, we listed 76 medicinal plants belonging to 39 families in alphabetical order with the parts used and the method of preparation according to their therapeutic use, which are used to treat 106 ailments.

**Conclusions:**

Many of the uses of medicinal plants mentioned in this survey are still under study. There is no doubt that this study will provide new data that could contribute to further pharmacological discoveries by identifying the active ingredients and their mechanism of effect by doing additional pharmacological work to confirm the alleged biological activities of these plants.

## Introduction

Traditional medicine (TM), as defined by the World Health Organization (WHO), is the sum total of the knowledge, skills and practices based on the theories, beliefs and experiences indigenous to different cultures, whether explicable or not, used in the maintenance of health as well as in the prevention, diagnosis, improvement or treatment of physical and mental illness (WHO 2002a, 2002b). Some TM systems are supported by huge volumes of literature and records of the theoretical concepts and practical skills; others pass down from generation to generation through verbal teaching. To date, in some parts of the world, the majority of the population continue to rely on their own TM to meet their primary health care needs. When adopted outside of its traditional culture, TM is often referred as complementary and alternative medicine (CAM) (Che et al. 2017).

Although modern medicine is currently available in many developing countries, large proportions of the population in these countries still rely to a large extent on traditional practitioners and medicinal plants for therapeutic purposes. TM is often the first choice for providing primary health care in developing countries, and the WHO estimates that more than 80% of healthcare needs in these countries are met by traditional health care practices, being the cheapest and most accessible (WHO 2002a, 2002b, 2004, 2005).

The WHO pays special attention to TM and CAM. Resolution (no. WHA13-6) issued by the World Health Assembly (WHA) in 2009 emphasized the need to update the global TM strategy (WHO 2009), so the WHO issued its strategy for traditional (folk medicine) 2014–2023 (WHO 2013).

The United Nations Educational Scientific and Cultural Organization (UNESCO) works to document the medical heritage of peoples within its documentation of the living intangible cultural heritage of peoples and civilizations, through the documentation of TM practices that were included in the UNESCO convention (2003), the convention on biological diversity (1992) and the UNESCO universal declaration on cultural diversity (2001) and the United Nations declaration on the rights of indigenous peoples (2007), and according to the meeting of the UNESCO International Bioethics Committee (IBC) working group on TM and its ethical effects in Paris (2010), it stressed the need to conduct studies that illustrate the use of TM in various regions around the world, and its evaluation in clinical practice and in research and policy (IBC 2013).

In 2017, the International Center for Information and Networks for Intangible Cultural Heritage in the Asia-Pacific region (ICHCAP) under the auspices of UNESCO, issued a book entitled Traditional Medicine in which the Syria Trust for Development in Section VII included TM in Syria (Falk 2017).

Ethno-botany is the scientific study of the relationships between people and plants. It was first coined in 1896 by the US botanist John Harshberger; however, the history of ethnobotany began long before that (Campbell et al. 2002; Amjad et al. 2015). It plays an important role in understanding the dynamic relationships between biological diversity and social and cultural systems (Husain et al. 2008; Amjad et al. 2013, 2015). Plants are essential for human beings as they provide food, and medicines (Alam et al. 2011; Ahmad et al. 2012).

Traditional Arabic medicine (TAM) is one of the famous traditional medical systems, which is occasionally called Unani medicine, Graeco-Arabic medicine, humoral medicine or Islamic medicine. The subject of TM in Syria has received little attention in the literature, and very little is known about the traditional medicinal substances used nowadays by the Syrian population to treat the most common diseases. Throughout ancient times in Syria, as part of the Levantine Nations (Bilad al-Sham), and other lands in the region, humans used various natural materials as sources of medicines (Jaddouh 2004). In the western countryside of Hama, there is a natural reserve for medicinal plants, which is called the Abu Qubais Protected area in Al-Ghab region (which protect the biodiversity rights of indigenous people and affiliated to the general commission for Al-Ghab administration and development), 509 plant species belonging to 72 families have been recorded (Al-Mahmoud and Al-Shater 2010).

For these reasons, the present investigation gathered the uses of medicinal plants in the centre region of Syria (Homs and Hama), as a supplement for a national survey, and documents the information concerning the uses of medicinal plants, which may serve as the basis of knowledge for a more intensive scientific research.

## Methods

### Study area

Syria is a country located on the east coast of the Mediterranean Sea in southwestern Asia. Syria is bounded by Turkey to the north, Iraq to the east and southeast, Jordan to the south and Lebanon to the southwest. The study area is the middle region of Syria including Homs Governorate and Hama Governorate.

This area has a Mediterranean climate with a long dry season from May to October. In the extreme northwest, there is some light summer rain. On the western region, summers are hot, with mean daily maximum temperatures ranging from low to mid 80 °F, while the mild winters have daily mean minimum reaching temperatures low level of 50 °F. Only above about 1500 m are the summers relatively cool. In inland the climate becomes arid, with colder winters and hotter summers. In the desert, at Tadmur maximum temperatures in the summer, temperature reaches averages in the ranges of upper 90s to low 100 °F, with extremes in the 110 °F. In rural areas, work takes place according to the seasonal rhythm of agriculture. Women generally share in much of the agricultural labour. Agriculture constitutes an important source of income, and fruits and vegetables including onions, olives and grapes. Commercially important forest plants include: pistachio, which is important for its oil-rich fruit, and plants such as olive trees, grapevines, apricot trees and cumin (Hamidé et al. 2021).

Historically, the ancient Palmyra, also called Tadmur, is an ancient city in south-central Syria (Homs Governorate). An oasis in the Syrian desert, Palmyra contains the monumental ruins of a great city that was one of the most important cultural centres of the ancient world, from the first to the second century according to World Heritage List (UNESCO 2021).

### Field work and data collection

Field surveys were conducted between December 2019 and January 2021, to document ethnobotanical information through oral interviews and designed semi-structured questionnaire. Thirty-five villages and 59 districts were visited for field research; 235 people were contacted and 151 of those with ethno-botanical experience agreed to become our informants, including 35 local herbalists (Tabib Arabi) and 10 physicians (who hold a general medicine specialty). The queries were repeatedly made to increase the reliability of the data; interviews with the men were usually carried out in the ‘Mukhtar’ house where they come together, and with women in their homes, bazaars and gardens. The Syria Trust for Development (which is a national development organization, and has a program called ‘Mashrouie’ that runs innovative microcredit programs that encourage economic growth in disadvantaged areas) helped us in data collecting. The information gathered during the present study included socio-demographic characteristics of the interviewed informants (age, gender) and ethnopharmacological information, including the local and scientific name of the species, local names, plant parts used, modes of use, conservation method, administration mode and toxicity, all documented data were then translated into English and Latin. Information that had been carried to the region from the outside and that was not used or confirmed was not included and recorded (Weckerle et al. 2018). During the interviews, questions about the following were asked to the participants:

1. Name and surname
2. Age and sex
3. Educational level
4. Are plants collected in your region?
5. Do you have any contact with plants?
6. Can you show the plants you use in your region?
7. Can you tell the names of local plants you use in your region?
8. In which season do you collect the plants you use in your region?
9. When collecting plant, which parts of the plant do you collect and how do you collect them?
10. Which parts of the plants do you use? (flowers, fruits, leaves, roots, tubers, young shoots, branches, stems, aerial parts, etc.).
11. How do you prepare and administrate the plants’ parts?
12. How did you diagnose the disease (by physician, by traditional healers, self-treating)?
13. How did you check the effectiveness of the treatment (disappearance of symptoms, by laboratory analysis, other methods)?

Only the medication of single herbs was included, while mixtures and multiple recipes were disregarded. The answers that were given with doubt to the questions above were not recorded, and we adopted the information that gave 40% frequency in the collected data, and the data that gave less than that were neglected.

Information about diseases diagnosed specifically by physicians and traditional healers was approved, while self-treating diseases were referred to as auxiliary in treatment, nutritional uses were clearly indicated, and some plants were used to prevent disease and improve public health. The information about diseases was divided into three groups when analysing the data (group A: acute diseases, group B: chronic diseases, group C: simple symptoms and signs), and the focus was only on groups B and C.

### Taxonomic identification of the species

Medicinal plants being mentioned by the Informants were recorded with local names and photographed. Each reported medicinal plant species was gathered, compressed, dehydrated, mounted on herbarium sheets, and identified; the taxonomic identity of the plants was confirmed by Prof. Abdel Aleem Bello (PhD/Botanical Taxonomy) and Dr. Bayan Tiba (PhD/Botanical Taxonomy) Aleppo University. As far as possible, the name of the plants was updated by consulting the latest literature; generic and species names followed the plant list (http://www.theplantlist.org). All voucher specimens have been preserved during documentation and deposited in the Damascus University, Faculty of Pharmacy, Pharmacognosy Labs Herbarium for future reference, serial numbers were taken from (1^ch^ to 76^ch^) according to its alphabetical order.

### Ethics approval and consent to participate

The study was approved by the Ethics Committee of the University of Damascus. Before beginning data collection, we obtained verbal informed permission in each case site-wide and then individually before each interview. We also informed informants that it was an academic project and that the investigation was for research purposes only, and not for any financial or other benefits. Informants provided verbal informed consent to participate in this study; they were free to withdraw their information at any time. These informants freely accepted the interview. All steps of research are consistent with Ethnobiology Code of Ethics (ISE 2006).

### Data analysis

The data collected through interviews of the informants were classified and examined with the statistical program IBM^®^ SPSS^®^ Statistics 26 (IBM, Armonk, NY), to determine the proportions of different variables such as ethnopharmacological data. Quantitative value indices were analysed using different statistical quantitative tools, i.e., the use reports (UR) of a species, and use value (UV) (Chaachouaya et al. 2021).

### Use value and use reports

The UR of a species or its importance in the culture of a community is denoted by its mentioning rate or its mention frequency by informants. The UR of the species of plants being utilized was evaluated using the formula (Tenté et al 2012):

UR = Ni/n

where Ni is the number of times a particular species was mentioned; *n* is the total number of times that all species were mentioned.

The UV of recorded medicinal plants was determined by applying the following formula (Tabuti et al. 2003):

UV=∑ URi/N

where ∑ URi is the total number of UR per plants; *N* is the total of interviewees questioned for given medicinal species.

The UV rate will be more important if there are several useful records for a species, implying that the plant is significant, whereas they will be near 0 if there are few reports compared to its use (Yaseen 2015; Chaachouaya et al. 2021).

## Results

### Demographic data of informants

In total, 151 local inhabitants of 35 villages and 59 districts were selected based on their experience in traditional uses of plants. Table 1 shows the age and gender wise distribution. All of them were interrogated using semi-structured questionnaires. Generally in Syria, both genders were interested in herbal medicines.

**Table 1.** Age and gender distribution.

Age group	Gender	No. of informants
Over the age 65	Male	20
	Female	12
50–65	Male	60
	Female	25
35–50	Male	19
	Female	15
Total	151	

### Ethnobotanical and ethnomedicinal uses of plant species

The study area is considered one of the Syrian areas rich in medicinal plants. In the east of it there is a desert (badia), which is dominated by desert and thorny plants, and in the west of it, aromatic plants are spread in the mountains near the eastern coast of the Mediterranean Sea. It is a fact that people in some of these rural areas suffer from poverty, so they depend a lot on folk remedies, and folk healers in these areas provide their expertise at small costs, because the medicinal herbs are cheaper than chemical medicines, and most of the medicinal recipes are available around. However, a large portion of the uses of medicinal plants mentioned in the research are still under study.

A total of 76 medicinal plant species (57.9% are wild and 42.1% are cultivated plants) belonging to 39 families are recorded in the present study; they are being used for a variety of purposes by native people. The detailed inventory is provided in Table 2, which includes botanical names, followed by local name, family and ethnobotanical uses.

**Table 2.** Ethnobotanical uses of plant species according to ethnomedicinal survey of central region in Syria (Homs and Hama).

Name of species	Common name (Arabic language)	Family	Part used	UV	Ethnobotanical uses
*Achillea santolina* L. W* 1^ch^**	قيصوم (Kaisoom)	Asteraceae	L	0.529	An infusion of the leaves is used internally for fever, common cold, absence of menstruation, dysentery, diarrhoea, loss of appetite, gastrointestinal (GI) tract discomfort, and to induce sweating.
*Allium cepa* L. C* 2^ch^**	البصل (Al-basal)	Liliaceae	Bb	0.827	A juice of the fresh bulb is used internally for cough, asthma attacks, typhoid, and the roasted bulb is eaten for diabetes, A juice of the fresh bulb is used externally for whooping cough, back pain, neck pain and warts.
*Allium sativum* L. C* 3^ch^**	ثوم (Thoum)	Liliaceae	Bb	0.894	Fresh bulb is used internally for hypertension treatment, vermifuge, diseases of the gastrointestinal tract, and urinary tract infection, relieve atherosclerosis, diabetes, anti-inflammatory, the bulb oil is used externally for rheumatism, dandruff, scabies, fungi and treating insect bites.
*Ammi visnaga* L. Lam. W* 4^ch^**	خلة سورية (Khelleh)	Apiaceae	Ps, Sd	0.728	Pedicles of this plant used to clean the teeth, and seeds decoction used internally as a diuretic, antispasmodic and for bladder stones. Also it is used as a smooth muscle relaxant for asthma, and whooping cough.
*Artemisia herba-alba* Asso. W* 5^ch^**	الشيح الأبيض (Shieh abiad)	Asteraceae	L	0.496	An infusion of leaves is used internally for cancer, nerve system disorders, heart diseases, diabetes, to increase appetite, and anthelmintic.
*Asparagus officinalis* L. W* 6^ch^**	هليون، عربيش (Heliun)	Asparagaceae	R, Sh	0.331	Cooked roots and shoots are used internally for urinary tract diseases, lowering blood pressure, analgesic and anti-inflammatory.
*Avena sativa* L. C* 7^ch^**	شوفان (Shofan)	Poaceae	Sd	0.436	The bran husk of seed is a mealy nutritive herb used internally for weight reduction, and constipation, the whole grinded seeds are used internally for diabetes, hypercholesterolaemia, diuretic and antispasmodic.
*Borago officinalis* L. W* 8^ch^**	لسان الثور، بلغصون (Lisan athaur)	Boraginaceae	L	0.662	An infusion of leaves is used internally for treatments for infections, respiratory complaints, depression, arthritis, asthma and the fresh leaves are used as mealy nutritive herb and diuretic.
*Arum maculatum* L. W* 9^ch^**	لوف (Louf)	Araceae	L	0.529	Cooked leaves are used internally for digestion disorders treatment, asthma treatment, bacterial infection, anthelmintic and liver tonic.
*Capparis spinosa* L. W* 10^ch^**	قبار (Cabar)	Capparaceae	Sh, R	0.43	Young shoots pickled either in vinegar or preserved in granular salt are used internally for diseases of the nervous system, back pain, liver diseases, the fresh roots are used externally for back pain and rheumatism.
*Carissa edulis* (Forssk.) Vahl W* 11^ch^**	عيرون (Airon)	Apocynaceae	R	0.19	A decoction of root is used externally for lichen disease.
*Carthamus tinctorius* L. W* 12^ch^**	عصفر (Isfer)	Asteraceae	Sd	0.099	A seed oil is used internally to prevent heart disease, dysmenorrhoea, amenorrhoea, postpartum abdominal pain, pain of joints.
*Celosia cristata* L. W* 13^ch^**	عرف الديك، القطيفة (Eurif aldiyk)	Amaranthaceae	L, Sd	0.125	The leaves decoction is used internally as an antidiarrheal, seed decoction is used internally as a laxative, in cases of cough, and dysarthria urination, and for pain.
*Centaurea calcitrapa* L. W* 14^ch^**	كليبة، مرار (Kllybeh)	Asteraceae	L	0.629	The fresh leaves are used internally as appetite enhancer, and for diarrhoea, the cooked leaves are edible as special traditional Syrian recipe known as Syrian salad (saleeg).
*Chrozophora tinctoria* L. A.Juss. W* 15^ch^**	غبيرة، صفيرة (Ghubyreh)	Euphorbiaceae	L	0.132	The fresh leaves are used internally as diuretic, and for kidney stones.
*Cichorium intybus* L. W* 16^ch^**	هندباء (Hinduba)	Asteraceae	L, R	0.92	An infusion of leaves is used internally for improving immunity, protecting the heart, and for cancer, and eye inflammation, the fresh leaves are used as diuretic, laxative and slimming, the roasted of roots for liver diseases, the leaves are edible as special salad.
*Citrus limon* L. Osbeck C* 17^ch^**	ليمون حامض (Limun hamd)	Rutaceae	F, Pe	0.596	Fresh juice of fruits is used internally for flu, common cold, the dried peels of fruits are used for diseases of stomach, intestine and urinary tract.
*Convolvulus althaeoides* L. W* 18^ch^**	مداد (Maddad)	Convolvulaceae	L	0.099	An infusion of leaves is used internally as diuretic, and for kidney stone.
*Coriandrum sativum* L. C* 19^ch^**	كزبرة (Kuzbara)	Apiaceae	Sd, L	0.86	A decoction of seeds and leaves is used internally for intestinal inflammation, weight loss and intestinal gas, treating narrowed arteries, diabetes.
*Crataegus azarolus* L. W* 20^ch^**	زعرور (Zaaroor)	Rosaceae	F	0.76	A decoction of fruits is used internally for cardiovascular diseases, hypertension, sexual weakness, cancer and diabetes.
*Cyperus rotundus* L. W* 21^ch^**	السعد (Sued)	Cyperaceae	T, Sd	0.112	A decoction of tuber part is used internally for digestion, bedwetting, diarrhoea, diabetes, inflammation and gastrointestinal disorder, the oil of seeds is used externally for permanent hair remove and for burns.
*Dittrichia viscosa* (L.) Greuter W* 32^ch^**	الطيون (At-tayun)	Asteraceae	L, Fl	0.841	A decoction of leaves is used externally for burns, wounds, cutaneous leishmaniasis and the oil of the flowers is prepared in olive oil to use topically, and it is used internally for anaemia, respiratory problems, ulcers of the gums, diarrhoea.
*Ecballium elaterium* L. A.Rich. W* 22^ch^**	عجور الجقل (Ajour eljacal)	Cucurbitaceae	F	0.37	Fresh juice of fruits is used for liver diseases, jaundice and sinusitis by nasal administration (just one drop of juice is inhaled in each nostril), and it is used externally for eczema.
*Eremostachys laciniata* L. Bunge W* 23^ch^**	حزنبل (Hoznobul)	Lamiaceae	R, Fl	0.21	A decoction of root and flower is used internally for allergy, headache and liver diseases, sedative.
*Eruca sativa* Mill. C* 24^ch^**	جرجير (Jarjeer)	Brassicaceae	L	0.807	Fresh leaves are used internally for sexual weakness and blood purification, diabetes, anti-toxicant, oil from seeds is used for hair tonic, burns, skin lesions, the leaves are edible as special salad.
*Eryngium creticum* Lam. W* 25^ch^**	قرصعنة (Korsanneh)	Apiaceae	R, Fl, Ap	0.688	A decoction of root and flower is used internally for liver diseases, poisonous, insect bites, anaemia and infertility problems, the young aerial parts are edible as special salad.
*Eucalyptus globulus* Labill. C* 26^ch^**	كينا الشام (Kenna el-Sham)	Myrtaceae	L	0.92	An infusion of leaves is used externally as inhalation for respiratory diseases.
*Ficus carica* L. C* 27^ch^**	التين (At-tīn)	Moraceae	F, Lx	0.668	The dried fruits externally for wounds, the latex is used externally for warts, the decoction is used internally for catarrh and bronchitis, and for diabetes, hypertriglyceridaemia, laxative.
*Foeniculum vulgare* Mill. W* 28^ch^**	شمرة (Shamra)	Apiaceae	Sd	0.788	An infusion of seeds is used internally as carminative, digestive, lactogogue and diuretic and in treating of respiratory and gastrointestinal disorders.
*Fraxinus syriaca* Boiss. W* 29^ch^**	دردار (Dardar)	Oleaceae	F	0.907	An infusion of leaves is used internally for facilitate digestion, treat tracheitis, strengthening the immune system, the cooked leaves known as traditional Syrian food in Salamiyah (is a city and district in western Syria, in the Hama Governorate).
*Glycyrrhiza glabra* L. C* 30^ch^**	عرقسوس (Erqsus)	Fabaceae	R	0.854	An infusion of roots is used internally for fever, ulcer, kidney diseases and asthma, rheumatism, its syrup famous as traditional Syrian drink during the holy month of Ramadan (fasting month) as anti-thirst.
*Hypericum triquetrifolium* Turra. W* 31^ch^**	عرن (Aran)	Hypericaceae	Fl	0.682	An infusion of flowers is used internally as anxiolytic, and antidepressant.
*Juglans regia* L. C* 33^ch^**	جوز (Jauz)	Juglandaceae	Sd, L, F	0.768	A decoction of seeds and leaves is used internally for sexual impotency, blood purification, lymph gland enlargement and bleeding, and topically is used for scrofula disease, sores, blisters, the fresh fruits are used for poor memory, strengthen immunity.
*Juniperus communis* L. et Viv. W* 34^ch^**	عرعر (Arâr)	Cupressaceae	F, Co	0.715	A decoction of cones and fruits is used internally for rheumatism, paralysis, tuberculosis, anaemia, diuretic and for urinary tract infection.
*Lepidium sativum* L. C* 35^ch^**	رشاد (Rashad)	Brassicaceae	L, Sd	0.867	A decoction of leaves is used internally for kidney disorders, diuretic, kidney stone, to increase breast milk in female, and tonic, it used to regulate the menstrual cycle in women, and to reduce blood sugar for diabetics, and triglyceride levels and blood cholesterol level, the oil of seed is used externally as hair tonic.
*Linum usitatissimum* L. C* 36^ch^**	كتان (Kettan)	Linaceae	S	0.662	Seeds oil is used internally as laxative and for obesity. The seeds are used in treatment of urinary tract infections and hypertriglyceridaemia.
*Malus trilobata* (Labill. ex Poir.) C.K.Schneid. C* 37^ch^**	تفاح (Tofah)	Rosaceae	F	0.576	Apple cider vinegar is used internally for slimming and reducing blood lipids, externally, it is used to treat skin diseases, remove corns, and as an antiseptic, and to treat lichen.
*Malva sylvestris* L. W* 38^ch^**	خبيزة (Khubbeizeh)	Malvaceae	L, Fl	0.682	A decoction of leaves and flowers is used internally for cough as expectorant, sedative for sleep problems, digestion problems and mouth sores, and externally for skin diseases.
*Matricaria aurea* (Loefl.) Sch.Bip W* 39^ch^**	بابونج ذهبي (Babunaj dahabi)	Asteraceae	Ca	0.649	The capitulum decoction or infusion is used internally for fever, coughing and heart diseases, chest pain, headache and kidney stone, and its used externally to treat skin infections, burns, wounds, eczema.
*Matricaria chamomilla* L. (Loefl) Sch.Bip. C* 40^ch^**	بابونج عادي (Babunaj aadi)	Asteraceae	F	0.788	The flower decoction or infusion is used internally for chest diseases, treatment of stomach ache, diabetes.
*Melissa officinalis* L. W* 41^ch^**	مليسة (Mellisa)	Lamiaceae	L	0.701	A decoction of leaves is used internally as carminative, antispasmodic, depression, anxiety, cough, respiratory infection.
*Mentha pulegium* L. C* 42^ch^**	نعناع (Nana)	Lamiaceae	L	0.741	A decoction of leaves is used internally as antiseptic, menstrual complaints, diaphoretic, sedative, itching, common cold, respiratory tract disorder.
*Micromeria myrtifolia* Boiss. & Hohen. W* 43^ch^**	زوفا (Zufa)	Lamiaceae	L	0.814	A decoction of leaves is used externally for wounds, sores, skin diseases and its used internally for colic and cold, heart diseases, digestive system and asthma.
*Myrtus communis* L. W* 44^ch^**	آس، حنبلاس (Aass)	Myrtaceae	L	0.701	A decoction of leaves is used internally for diarrhoea, respiratory tract diseases and externally for haemorrhoids.
*Nigella sativa* L. C* 45^ch^**	حبة البركة (Habbet barakeh)	Ranunculaceae	Sd	0.768	Seeds are ground finely and used internally as expectorant, carminative, impotency in male, antispasmodic, hypoglycaemic, oil used externally for skin diseases, hair growth and orally to strengthen the body's immunity.
*Olea europaea* L. C* 46^ch^**	زيتون (Zaitoon)	Oleaceae	L, F	0.9	A decoction of leaves is used internally for diabetes, high blood pressure and slimming. A fruit's oil is used for coughing, vasodilator, laxative, hyperacidity and stones in kidney, and its used externally for skin diseases.
*Opuntia ficus-indica* L. Mill. W* 47^ch^**	تين الصبار (Tīn alsabr)	Cactaceae	F, L	0.682	Fresh fruits and leaves extract or juice are used internally as laxative, anti-inflammatory, carminative, digestive and are used externally for sun burns, skin care, burn, wound.
*Origanum syriacum* L. C* 48^ch^**	زوبع، مردقوش (Zoba'a)	Lamiaceae	Ap	0.721	A decoction of aerial parts is used internally for catarrh, carminative, diuretic, headache, rheumatism, antiseptic, neck stiffness, stomach cramps, stomach discomfort, indigestion, cholesterol reduction.
*Papaver rhoeas* L. W* 49^ch^**	شقائق النعمان (Shakaek alnuman)	Papaveraceae	Fl	0.509	A decoction of flowers is used internally for whooping cough, headache and has hypnotic effect, analgesic, as relieves stress.
*Paronychia argentea* Lam. W* 50^ch^**	زهرة الماسة (Zahret el-maseh)	Caryophyllaceae	Fl	0.668	An infusion of flowers is used internally as diuretic, and for kidney stone.
*Petroselinum sativum* Hoffm. C* 51^ch^**	بقدونس (Bakdoones)	Apiaceae	Fl	0.655	An infusion of flowers is used internally for anaemia, and calms nerves, and anti-toxic, and for inflammation, and as liver tonic, also it is used externally for treating bruises, insect bites and rough skin.
*Pistacia atlantica* Desf. W* 52^ch^**	بطم (Batm)	Anacardiaceae	F, G	0.225	The fruits of the mastic are called green ivory in the Syrian countryside, fresh green fruits are used internally for treating the liver diseases, tumours, joint diseases, and colds and headaches, joint pain, gum tree is used externally in treating scabies, wounds and fungal diseases, the traditional Jarmashi bread with the fruits of the mastic, where the green fruits give it a delicious taste and gives great energy and vitality, Mastic tree keeps poisonous insects away.
*Pistacia vera* L. C* 53^ch^**	فستق حلبي (Fustuk Halabi)	Anacardiaceae	F, Pe, G	0.754	The fruits are nutritious, and the peels of fruits and gum of the tree are used externally for cases of eczema, dermatitis and psoriasis.
*Plumbago europaea* L. W* 54^ch^**	خامشة (Khamisheh)	Plumbaginaceae	Fl	0.066	An infusion of flowers is used externally for treating alopecia and psoriasis.
*Portulaca oleracea* L. C* 55^ch^**	بقلة (Albaqlata)	Portulacaceae	Ap	0.715	An infusion of flowering herbs is used internally for kidney disorders and improve digestion, prevention of heart disease and cancer, and for weight reduction, stomach diseases and bone strengthening.
*Prunus armeniaca* L. C* 56^ch^**	مشمش (Mushamash)	Rosaceae	F	0.582	Dried fruits are used internally to strengthen the body's immunity.
*Punica granatum* L. C* 57^ch^**	رمان (Rumman)	Punicaceae	F	0.622	The fruit juice is used internally for mouth sores, cough, malabsorption syndrome, hypercholesterolaemia, the bark is used as anthelmintic, and for diarrhoea, amoebic dysentery, antibacterial, ulcer.
*Quercus calliprinos* Webb. W* 58^ch^**	بلوط (Ballot)	Fagaceae	F, Bk	0.298	A decoction of fruits and bark is used internally as anti-bleeding and pain reliever, and helps in digestion, blood purification, coughing, and it is used externally for eczema treatment.
*Quercus ithaburensis* Decne. W* 59^ch^**	سنديان (Cendyan)	Fagaceae	S, Bk	0.185	A decoction of stem and bark and fruit is used internally for cancer, fever, bed wetting, high blood pressure and ulcer.
*Raphanus raphanistrum* L. W* 60^ch^**	فجل بري (Fejel barri)	Brassicaceae	R	0.682	A decoction of roots is used internally as diuretic, anti-cancer, and for rheumatism, and the dried roots is used for liver diseases.
*Rhus tripartita* (Ucria) Grande W* 61^ch^**	عرينة (Aryneh)	Anacardiaceae	W	0.086	A decoction of whole plant is used internally as diuretic, and for kidney stone.
*Rosa*×*damascena* Herrm C* 62^ch^**	وردة شامية (Wardeh shamieh)	Rosaceae	Fl	0.582	Rose water is used externally for skin diseases, and skin care, the petals are used internally for cough, and laxative, and to treat chest infection. UNESCO has inscribed the element of the Damascus rose and associated heritage practices and craftsmanship on the UNESCO intangible cultural heritage of humanity list for its importance in making essential oils which can be used in traditional medicine (UNESCO 2019).
*Rosmarinus officinalis* L. C* 63^ch^**	اكليل الجبل (Eklil aljabal)	Lamiaceae	L	0.834	An infusion of leaves is used internally for respiratory diseases, heart disorders, to enhance memory, enhance the body's immunity, treating headache and as antidepressant.
*Rubus fruticosus* G.N.Jones W* 64^ch^**	توت العليق (Toot alealiq)	Rosaceae	F, R	0.549	An infusion of fruits and root is used internally for kidney stone, glycaemic, atherosclerosis, hypotensive and anticoagulant.
*Rumex obtusifolius* L. W* 65^ch^**	حميضة (Homedah)	Polygonaceae	L	0.523	An infusion of leaves is used internally for mouth ulcers, for anaemia, adjust sugar, improve blood circulation, the cooked leaves are edible as special traditional Syrian recipe known as Syrian salad (saleeg).
*Salvia fruticosa* Mill. C* 66^ch^**	ميرمية (Meiramiea)	Lamiaceae	L	0.609	An infusion of leaves is used internally for stomach and colon disorders, regulator and sterilizer for uterine diseases in women, intestinal antiseptic, diuretic, diabetes.
*Sarcopoterium spinosum* L. Spach W* 67^ch^**	بلان (Ballan)	Rosaceae	R	0.45	A decoction of roots is used topically for treating joints and spinal disc, a decoction of seeds is used internally for haemorrhoids, diabetes.
*Sorghum halepense* L. Pers. W* 68^ch^**	حليان (Halyan)	Poaceae	W	0.139	It’s a poison plant, but it is used internally for the treatment of urinary tract disorders, diuretic, kidney stone.
*Thymus syriacus* Boiss. C* 69^ch^**	زعتر (Zaatar)	Lamiaceae	L	0.695	An infusion of leaves is used internally as anti-cough, bronchitis, carminative, antispasmodic, anthelmintic and diabetes.
*Trigonella foenum-graecum* L. C* 70^ch^**	حلبة (Halbeh)	Fabaceae	Sd	0.509	A decoction of seeds is used internally as antitussive, hypercholesterolaemia, galactagogue, atherosclerosis, diabetes, blood pressure, heartburn, cold, inflammations, topical massage for joint and bone pain, kidney stone, and to gain weight, the oil of seed is used externally for breast enlargement.
*Triticum aestivum* L. C* 71^ch^**	حنطة (Hentah)	Poaceae	Sd	0.629	An infusion of seeds is used internally for constipation and obesity as the bran husk is used, it is used externally as compresses for skin itch.
*Urtica dioica* L. W* 72^ch^**	قريص (Kurras)	Urticaceae	Ap, Sd	0.496	An infusion of flowering part and seeds is used internally for rheumatism, joint and chest pain, anaemia, digestive diseases, kidney disease, gall and diuretic, cough and respiratory system, and for cessation of nosebleeds, the oil is used externally for cases of hair loss and cases of burns.
*Vicia faba* L. C* 73^ch^**	فول (Fool)	Fabaceae	Sd	0.529	Seeds are used internally as tonic, diuretic, beneficial to the heart, and for the menopause stage, diabetes.
*Vitis vinifera* L. C* 74^ch^**	عنب (Aleunab)	Vitaceae	F	0.668	Grapes fruits are popularly used internally as an optimal food to revitalize the body, especially the brain, and it is a good food for the heart, and is considered one of the most important sources of energy for muscles, besides the fruits are used for constipation and gout, the seeds are used as antihypertensives and reduce blood sugar, The seeds are used for menstrual cramps and period regulation, dried fruits called (Zabeb) are used to strengthen the body's immunity, and as general tonic, the seed oil is used externally as tonic for hair, and for skin diseases, the fruits vinegar also used internally for slimming.
*Xanthium strumarium* L. W* 75^ch^**	الحسك (Hasak)	Asteraceae	Sd	0.198	A decoction of seeds and leaves is used internally for infertility and impotency in males, galactagogue.
*Zea mays* L. C* 76^ch^**	شباشيل الذرة (Shabashel el-zurah)	Poaceae	St	0.642	An infusion of corn stylus is used internally for regulating blood sugar, reducing cholesterol, as diuretic for kidney stone, weight loss, gout, bedwetting.

Ap: aerial parts; Bk: bark; Br: branches; Bu: buds; Bb: bulb; Ca: capitulum; Co: cones; Fl: flower; F: fruit; G: gum; Lx: latex; L: leaves; Ps: pedicles; Pe: peel; Ph: phloem; P: pods; Rs: resin; Rm: rhizome; R: root; Sd: seeds; S: stem; St: stylus; T: tubers; W: whole plant.

* C: cultivated plants, W: wild plants.

** Herbarium no.

### Botanical families of plants used

**Figure 1.**
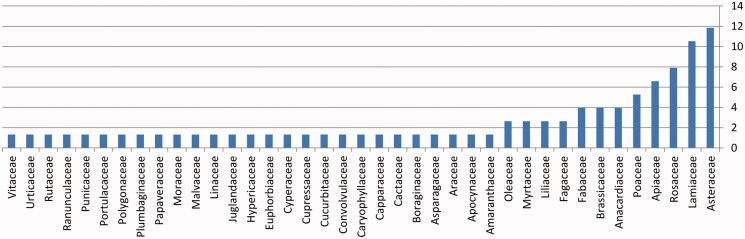
Plant families commonly used in ethnomedicinal survey of central region in Syria (Homs and Hama).

The most commonly mentioned family is Asteraceae (11.84%), followed by Lamiaceae (10.52%), then Rosaceae (7.89%) and Apiaceae (6.57%), Poaceae (5.26%), Anacardiaceae and Fabaceae (3.94%), Fagaceae, Liliaceae, Myrtaceae and Oleaceae (2.63%), then all the other families (1.31%) (Figure 1).

### Use value of the plants

Medicinal use plants (UV) are utilized to find the most frequently used plant species in the study area. Its value ranged from 0.066 to 0.92 (Table 2). The calculated results of UV showed that *Cichorium intybus* L., *Eucalyptus globulus* Labill. was ranked first (UV = 0.92) followed by *Fraxinus syriaca* Boiss. (UV = 0.907), *Olea europaea* L. (UV = 0.9), then *Allium sativum* L. (UV = 0.894), *Lepidium sativum* L. (UV = 0.867), *Coriandrum sativum* L. (UV = 0.86), *Glycyrrhiza glabra* L. (UV = 0.854), *Dittrichia viscosa* (L.) Greuter (UV = 0.841), while the lowest value was found for *Plumbago europaea* L. (UV = 0.066).

### Medicinal parts of the plant used

**Figure 2.**
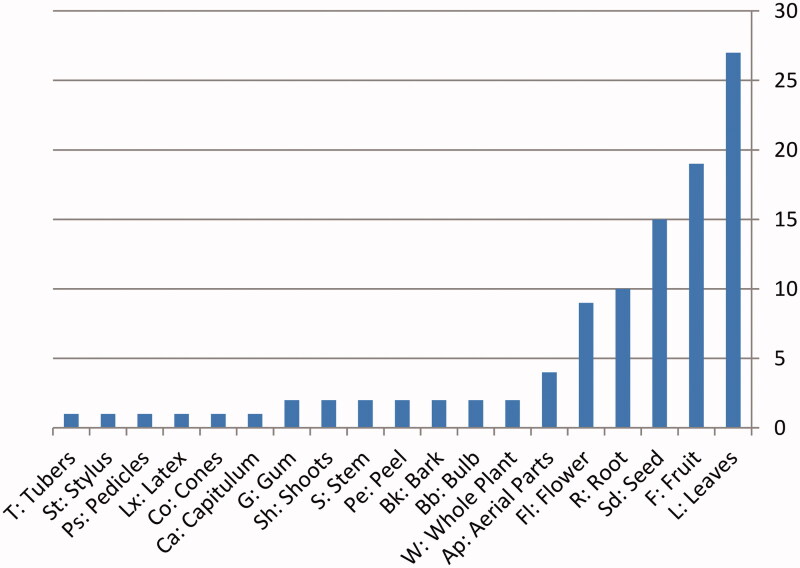
Medicinal parts of the plants used for ethnomedicinal purposes in the study.

The analysis of the ethnobotanical data showed that central region was best suited to the medicinal plant and rangeland. Ethnobotanical use categories showed that leaves were commonly used parts for making indigenous recipes (28.08%), followed by fruits (19.76%) and seeds (15.6%) then roots (10.4%), flowers (9.36%) and aerial parts (4.16%). Then, the others parts of plant are rarely used (Figure 2).

### Modes and conditions of medicine preparation

**Figure 3.**
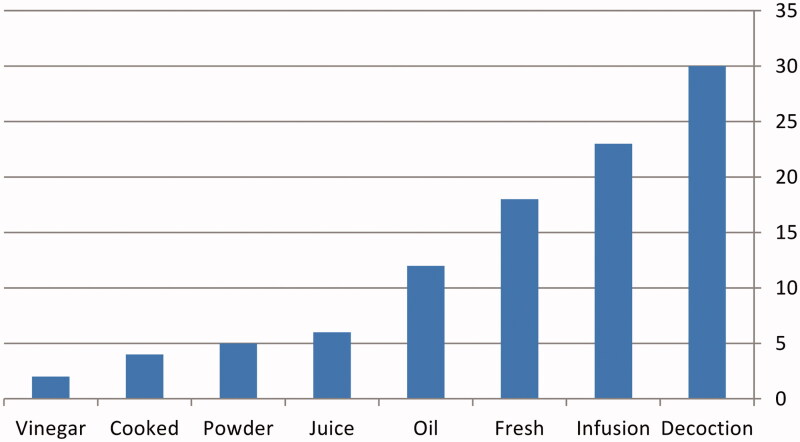
Modes of ethnomedicines preparation in Homs and Hama.

The analysis of the ethnobotanical data showed that the recipes in the most cases were obtained from single herb, but some of recipes were prepared together, and there is a famous local mixture called Damask tisane (zhourate Shamieh). A mode of TM preparation reported was a decoction (30%), followed by infusion (23%), and then by other method such as fresh herbs, juice, cooked, powder, vinegar and oils (47%) (Figure 3). Considering according to results, most of the plant preparations are used orally (Table 2).

### Ethnomedicinal information about treatment the different diseases

The results of questionnaires showed that 20% of the informants were diagnosed with their diseases by a doctor, and 45% were diagnosed with a conventional therapist, and 35% self-diagnosed their diseases, while the results of the questionnaires showed that the evaluation of the treatment by informants as following (58% relied on the disappearance of symptoms, and 24% through the results of laboratory analysis, 18% adopted other methods such as chest radiography, adopting the attending physician’s opinion and clinical observation of the improvement of skin diseases, and some of them depended on psychological comfort during treatment as evidence of improvement).

**Figure 4.**
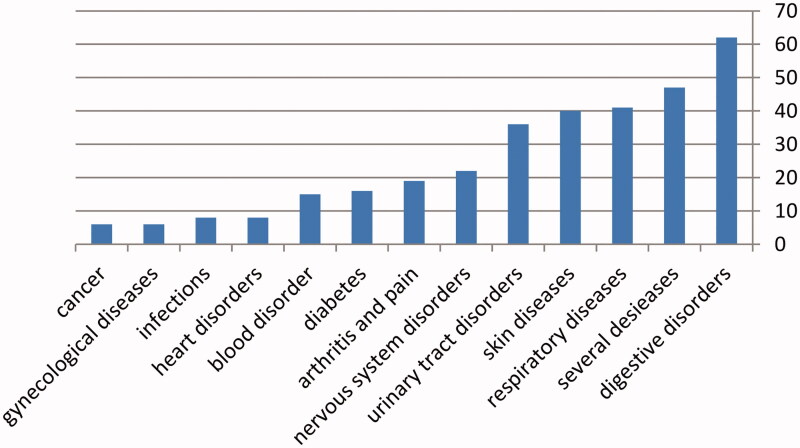
Ethno-medicinal information about treating different diseases related to central region in Syria (Homs and Hama).

Among these studied plants, 62 are used to treat digestive disorders, 41 for respiratory diseases, including asthma, bronchitis and coughs, 40 for skin diseases, 16 for diabetes, 36 for kidney and urinary tract disorders, 22 for nervous system disorders, six for enhance the body's immunity, two for haemorrhoids, five for fever, eight for heart disorders, five for infertility and impotence, six for treating several types of cancer, two for increasing breast milk production, five for losing weight, four for lowering cholesterol, and two for increasing weight, and six for anaemia, 15 for blood disorder, two anti-toxicant, 19 for arthritis and pain, one for typhoid disorder, eight for infections, six for gynaecological diseases, one for eye inflammation, two anti-toxicant and four for mouth sores. Many of them are still used today, especially those plants recommended for internal uses such as traditional medicinal teas, which mainly consist of remedies for obesity, weight loss, colds, colds, digestive disorders, abdominal pain, constipation and some skin diseases (Figure 4).

## Discussion

The use of TAM has spread to treat various diseases in Syria since ancient times. They are cost-effective with fewer side effects and are more suitable for long-term use compared with chemically synthesized medicines.

The ethnobotanical categories indicated that there is large use of medicinal herbs in the area of study, most of them are wild. There is an increased exploitation of medicinal plants by the local population, collectors and dealers of herbal medicines, in line with the demand from the pharmaceutical industry. This caused a sharp decrease in the occurrence and products of medicinal plants. Grazing, deforestation by cutting down trees for heating, and fires were mainly responsible for the reduction of medicinal plants. That is why the government is working on developing strategies to conserve wild plant diversity. Some people collect the medicinal plants for an income. They uproot and collect each part of the medicinal plants in non-scientific way. Thus, to date, a few articles devoted to TM of Syria, such as a study of folk medicine in Aleppo Governorate (Alachkar et al. 2011), and a study about the use of ‘Zahraa’ (Syrian traditional tisane) (Carmona et al. 2005), and a third one on the medicinal plants in Golan (Said et al. 2002), which is an occupied Syrian territory.

This research aspires to genuinely contribute in providing useful information on the conserving and sustaining the natural resources in the area.

The perspectives in the questionnaire were compared with other ethnomedicine studies in the countries surrounding Syria such as Lebanon (Taha et al. 2013), Jordan (Lev and Amar 2002; Al-Qura'n 2009), Palestine (Friedman et al. 1986; Kaileh et al. 2007), Iraq (Al-Douri 2000) and Turkey (Yeşilada et al. 1995; Sezik et al. 2001). Similarities in various traditional uses in Syria, Lebanon, Palestine and Jordan were observed. This is mainly due to the mutual history of these areas that were previously called Levantine Nations (Bilad al-Sham) (Lev 2002), and there is some similarity with a smaller number of folk uses both in Syria and Iraq, but there is a difference in the folk uses described between Syria and Turkey (Korkmaz et al. 2016; Yerebasan et al. 2020). We compared the folk uses of plants mentioned in related articles on ethnomedicine in these regions with the plants studied in our research to find out the extent of congruence or difference the uses of similar plants.

We did not record significant differences in phytomedicines consumption customs between interviewees of different religions. In general, phytomedicines consumption was often explained and justified by interviewees as family tradition. We did not detect any gender-related differences in phytomedicines consumption. There were no gender differences concerning the common traditional use of medicinal plants. The ethno-medicine data presented herein imply that medicinal plants are important as food and particularly as medicine (traditional healing) for various local people. While chemical medicinal treatments are becoming commonplace, traditional medications are still of huge importance in many rural, poor and remote places.

This study will undoubtedly provide new data that could contribute to further pharmacological discoveries by identifying the active ingredients and their mechanism of effect by doing a lot of pharmacological work to confirm the alleged biological activities of these plants, and the possibility of developing new pharmaceutical formulas cannot be excluded depending on Syrian medicinal plants and their folk uses; as the discovery of artemisinin from *Artemisia annua*, based on ethnobotanical information (Acton and Klayman 1985), serves as evidence that it is possible to find new and effective medicines using data from TM.

## Limitations

There is insufficient information about the pharmacokinetic efficacy of the medicinal plant species in this study. These herbs have reportedly and traditionally been used as adjuvant to relieve and treat some diseases.

## Conclusions

Many of the uses of medicinal plants mentioned in Syria are still under study. This study has been conducted with the aim to generate new concepts that could supplement pharmacological work with potentially further pharmacological discoveries; that is, by identifying the active ingredients and their mechanism of effect to confirm the alleged biological activities of these plants.

## Data Availability

Supplementary materials related to this article may be obtained from the authors upon request.
